# Research Note: Proteases effect on soybean meal: enzyme product and area of production differences on standardized ileal amino acids digestibility of broiler chickens

**DOI:** 10.1016/j.psj.2024.104493

**Published:** 2024-11-04

**Authors:** Hong Liu, Murtala Umar Faruk, Levy Teixeira, André Fávero, Sergio L. Vieira

**Affiliations:** a dsm-firmenich, Animal Nutrition and Health, R&D Center, Bazhou, China; b dsm-firmenich, Wurmisweg 576, 4303 Kaiseraugst, Switzerland; c Independent Consultant, Rua General Osorio, Garibaldi, RS 95720-000, Brazil; d Universidade Federal do Rio Grande do Sul, Porto Alegre, Brazil

**Keywords:** Protease, Soybean meal, Amino acid, Digestibility, Broiler

## Abstract

This study evaluated the effects of two protease products added to feeds having two soybean meal (SBM) sources (South and North of Brazil). A total of five hundred and sixty, 21 d male broiler chickens were allocated into 7 dietary treatments: a nitrogen-free diet (NFD) and other six diets prepared with a 40% replacement of the NFD with each SBM source. Two commercial protease products (RONOZYME® ProAct and ProAct 360^TM^) were added at 15,000 PROT and 30,000 NFP per kg feed, respectively. After 5-day diet adaptation, all birds were euthanized for ileal digesta collection. Results show that the North SBM sample was inherently higher (P < 0.05) in apparent ileal digestibility (AID) of dry matter (DM), nitrogen (N), and the standardized ileal amino acid digestibility (SIAAD) compared to South SBM sample. The DM digestibility and SIAAD of North SBM sample were not different from the un-treated control and protease treatments (P > 0.05, except Val and Ile, which had higher digestibility with ProAct 360). The DM digestibility and SIAAD from South SBM sample were increased (P < 0.05, except Met, Asp and Cys) with both supplemental proteases. Correlation analysis showed that AA digestibility of the two SBM samples were negatively associated with the effect of protease products. These data demonstrated that the SIAAD of SBM was influenced by SBM source with the South SBM sample being lower than that from the North while the protease effects were more evident in the SBM sample from South; responses of ProAct 360 were superior to ProAct. Exogenous proteases can enhance nutritional value of SBM, particularly for those with inherently low amino acid digestibility.

## Introduction

Soybean meal (SBM) is the predominant source of protein and amino acids in poultry feeds due to its high protein content and balanced amino acid profile. However, factors such as genetics, environmental and processing conditions as well as anti-nutritional factors can impact the nutritional value of SBM (Ibáñez et al., 2020). For example, the apparent metabolizable energy and digestible crude protein content of 55 SBM samples from four countries varied up to 375 kcal/kg and 3.7%, respectively (Ravindran et al., 2014). These variations may, in part, contribute to the inconsistencies in SBM nutritional values and the response to the supplemented exogenous enzymes in diets.

Proteases are enzymes that break down proteins into small peptides and amino acids, thus, making AA available for biological needs. Exogenous protease added to the feeds can complement the endogenously secreted proteases to support protein hydrolysis and increase the digestion of the dietary protein. However, as for any other enzyme, their action is dependent on the nature and availability of the substrate targeted. Knowledge of the nutritional value of feed ingredients (e,g., SBM) and their response to exogenous enzymes is of importance in the development of precision feed formulation and exogenous enzyme recommendations. Therefore, the objective of the study was to assess the SIAAD of two SBM samples from two geographic areas (South and North of Brazil) and their response to two different exogenous proteases (RONOZYME® ProAct and ProAct 360^TM^). Both protease products are commercially available with similar pH profile, intrinsic thermal stability, however, ProAct 360^TM^ has been reported to have higher specific activity at physiologically relevant pH of 4 and 5 (Cupi *et al.*, 2022).

## Materials and methods

Animal handling and management procedures were reviewed and approved by the Ethics and Research Committee of Universidade Federal do Rio Grande do Sul, Brazil.

### *Diets, Bird Management and Sample Collection*

Five hundred and sixty, day-old Cobb 500 male broilers obtained from a commercial hatchery were randomly allocated to pre-study battery cages, where they received a standard diet based on corn and SBM (AME, 3000 kcal/kg, crude protein, 208 g/kg) for 21 days. On day 21, birds were individually weighed and randomly allocated to one of 7 dietary treatments: a nitrogen-free diet (NFD) which was used for the estimation of endogenous amino acid losses, and other six diets prepared with a 40% replacement of the NFD with two SBM sources. The SBM samples were obtained from soybean producers with 200 kg of each either from Mato Grosso state (North of Brazil) or Rio Grande do Sul state (South of Brazil). Protease supplementation was done with two exogenous proteases at recommended doses (RONOZYME® ProAct and ProAct 360^TM^, DSM Nutritional Products AG, Kaiseraugst, Switzerland, at 15,000 PROT and 30,000 NFP per kg feed, respectively). One PROT or NFP unit is defined as the amount of enzyme that liberates 1 μmol para-nitroaniline (pNA) from 1 mmol/L (N-Suc-Ala-Ala-Pro-Phe pNA) substrate per minute at pH = 9.0 and at 37°C. The feeds were provided as mash and formulated according to the recommendations of Ravindran et al (2017) for the measurement of amino acid digestibility in feedstuffs in broilers (Table 1). Insoluble acid ash (Celite^TM^) was included at 1% as indigestible marker to enable the calculation of amino acid digestibility. Phytase at 1,000 FYT/kg (RONOZYME® HiPhos, DSM Nutritional Products AG, Kaiseraugst, Switzerland) was included in all diets as a commercial routine practice. There were either 16 (only for NFD and the two non-treated control diets) or 8 replicates cages of 7 birds each.

**Table 1:** Compositions of the experimental diets and soybean meal samples.

Ingredients (g/kg, as fed basis)	Nitrogen-free diet	NFD + SBM North	NFD + SBM South
Starch	200.80	-	-
Dextrose	650.00	502.70	502.70
Soybean meal	-	400.00	400.00
Corn cob (fiber)	50.00	-	-
Fat	50.00	40.00	40.00
Dicalcium phosphate	-	19.00	19.00
Monocalcium phosphate	1.90	-	-
Limestone	13.00	10.00	10.00
Sodium bicarbonate	7.50	2.00	2.00
Magnesium oxide	2.00	-	-
Potassium carbonate	2.60	-	-
Potassium chloride	2.90	-	-
Salt	4.50	2.00	2.00
Vitamin supplement^1^	1.30	1.30	1.30
Mineral supplement^2^	0.50	0.50	0.50
Choline chloride	2.50	2.00	2.00
Insoluble acid ash (Celite^TM^)	10.00	10.0	10.0
Phytase^3^	0.05	0.05	0.05
Total	1000.00	1000.00	1000.00
Chemical composition of the tested SBM (g/kg dry matter)			
Item		North of Brazil	South of Brazil
Dry matter		861.3	884.6
AMEn		2323	2246
Crude protein		550.0	512.0
Crude ash		70.8	71.3
Crude fat		18.2	30.7
Crude fiber		51.3	51.0
Total phosphorus		7.4	6.6
Phytic phosphorus		5.6	4.5
Lysine		33.2	31.9
Methionine		7.3	7.0
Cysteine		7.7	6.9
Threonine		21.6	20.5
Tryptophan		7.7	7.2
Valine		27.1	25.2
Isoleucine		26.2	24.5
Leucine		42.5	39.9
Phenylalanine		28.1	25.7
Histidine		14.0	13.2
Arginine		40.2	36.6

Abbreviations: NFD, nitrogen free diet; SBM, soybean meal.

1 Composition/kg of the feed: retinyl acetate, 14,300 IU; cholecalciferol, 5,200 IU; DL-α-tocopheryl acetate, 71.5 IU; thiamine, 2.99 mg; riboflavin, 9.1 mg; pyridoxine, 5.2 mg; D-calcium pantothenate, 15.6 mg; biotin, 0.325 mg; menadione, 3.9 mg; folic acid, 2.6 mg; niacinamide, 78 mg; cyanocobalamin, 32.5 mcg; Se (sodium selenite), 0.39 mg.

2 Composition per kg of feed: Mn (manganese oxide), 65.0 mg; Fe (ferrous sulfate), 50.0 mg; Zn (zinc sulfate), 65.0 mg; Cu (cupric sulfate), 10 mg; I (potassium iodide), 1 mg.

3 RONOZYME HiPhos GT with 20,000 FYT /g; the in-feed protease recovery with South SBM supplemented without or with ProAct and ProAct 360 were 1,248 FYT/kg, 1,673 FYT/kg and 1,480 FYT /kg while corresponding values with North SBM were at 1,191FYT/kg, 1,580 FYT/kg and 1,320 FYT/kg, respectively.

Temperature and lighting regimens were adjusted according to breed guidelines and birds’ health status was checked daily. Birds were allowed *ad libitum* access to feed and water. After 5-day of diet adaptation, all birds from cages were euthanized by electrical stunning. Ileal digesta content was collected from Meckel's diverticulum to 2 cm proximal to the ileo-cecal junction by gently flushing with distilled water and pooled by cage before frozen (at -20°C), the ileal samples were freeze-dried, ground to pass through a 0.5 mm sieve and stored at 4°C in airtight bags for chemical analysis.

### *Chemical analysis and calculations*

The SBM samples were collected from either South or North areas in Brazil and analyzed by FOSS DS2500 NIRs for chemical compositions (dry matter (DM), nitrogen corrected apparent metabolizable energy (AMEn), crude protein and amino acids, crude fat, crude ash, crude fiber, phosphorus (P) and phytate P). The feed and ileal digesta samples were analyzed for DM, and insoluble acid ash according to Joslyn (1970). N was determined by combustion method (method 968.06; AOAC International, 2006). The amino acid content was analyzed using high-performance liquid chromatography (HPLC), according to the methodology described by White et al (1986) and Hagen et al (1989). Calculations of the standardized ileal digestibility of amino acids were performed as proposed by Ravindran at al. (2017).

### *Statistical analysis*

Data were analyzed using the Fit mode platform in JMP version 16.0 (SAS Institute, Cary, NC, US). Outliers were excluded with the criteria with those values exceed means ± three times standard deviation. The statistical model included effects of SBM source, protease supplementation and their interactions. Cage served as the experimental unit. The correlation between the inherent SIAAD of the two SBM samples and the percentage effect of protease was performed using Fit Y by X platform. Statistically significance was set at *P* < 0.05.

## Results and discussion

The chemical composition of the SBM samples is presented in Table 1. The North SBM sample was slightly higher in crude protein (550 vs 512 g/kg) and some essential amino acids, as well as phytate P content, but lower in dry matter (861.3 vs 884.6 g/kg) and crude fat content (18.2 vs 30.7 g/kg) as compared to the South SBM sample. All other evaluations were similar between the two SBM samples. Phytase activity in feed samples ranged from 119% to 167% of expected values exhibiting some variability which was likely due to sampling and assay error and the actual enzyme activity is usually higher than it is claimed. The protease activity was not detected in the non-treated controls. The in-feed protease recovery in South SBM supplemented with ProAct and ProAct 360 were 13,610 PROT/kg and 32,013 NFP/kg, and corresponding values with North SBM stands at 12,710 PROT/kg and 22,091 NFP/kg, respectively. The initial body weight (d 21), feed intake and body weight gain during the 5-d period were not differed among treatments (data not shown).

Effects of SBM sources and protease supplementation on the apparent ileal digestibility (AID) of DM and N, and SIAAD are presented in Table 2. Overall, the North SBM samples was inherently higher (P < 0.05) in AID of DM (75.4% vs 71.1%) and N (81.5% vs 80.0%), and SIAAD (84.6% vs 79.7% for total AA) compared to the South SBM sample. It's in agreement with the findings by Stefanello et al (2016) who evaluated the amino acid digestibility of two SBM samples from the same geographic areas within Brazil. These results indicate that the nutritional quality of SBM is influenced either by geographic location, as well as environmental and processing conditions as mentioned. Both protease products increased AID of DM and SID of most AA (+3.8% and +6.6% for total AA with ProAct and ProAct 360, respectively) in the South SBM sample except Met, Asp, Cys, whereas only the digestibility of Val (+3.0%) and Ile (+2.8%) was increased by ProAct 360 and no clear evidence was noted for ProAct in the North SBM sample, resulting in interactions (P < 0.05) between SBM source and protease product for the digestibility of DM and most AA. Improvements in AA digestibility of SBM by exogenous proteases have been shown by Stefanello et al (2016) and Liu et al (2024). The discrepancy in enzyme response is likely attributed to the differences in the chemical compositions between the two SBM samples and the South SBM sample with lower inherent SIAAD was more readily for an enzyme response as compared to the sample from North. In the current study, the lack of protease effects on N digestibility was not in line with the digestibility results of total AA. In addition, correlation analysis showed that there was a downward quadratic correlation between the inherent AA digestibility of the two SBM samples and the effects of protease products on the same (data not shown). Similarly, Cowieson and Roos (2014) reported that the effect of exogenous proteases on N and AA digestibility was negatively associated with the inherent digestibility in the control diet. However, in the study by Stefanello et al (2016) there was a lack of interaction between SBM sources and protease supplementation, and this could be attributed to the differences in chemical characteristics of SBM sources, such as KOH solubility, urease activity and trypsin inhibitor contents, which were not measured in the current study. It is worth noting that ProAct 360 outperformed ProAct in terms of improving SIAAD in both SBM samples, indicating heterogeneity of two proteases in their proteolytic activity. The product ProAct 360 is a second generation of endopeptidase that can lead to a fast hydrolysis of protein chain. It is of interest that the digestibility results from the South SBM sample when added with supplemental ProAct 360 was equal or numerically superior to that in non-treated North SBM sample. This highlights improvements in economic returns with the supplemental protease when SBM with a lower amino acid digestibility is used.

**Table 2:** Effect of soybean meal origin and protease supplementation on apparent ileal digestibility of dry matter and nitrogen, and standardized ileal amino acid digestibility in broiler chickens.

		Apparent ileal digestibility coefficients, %		Standardized ileal digestibility coefficients (%)																	
SBM source	Protease	Dry matter	Nitrogen	His	Arg	Thr	Val	Met	Ile	Leu	Phe	Lys	Asp	Glu	Ser	Gly	Ala	Pro	Tyr	Cys	Total amino acids
North Brazil	-	75.4^ab^	81.7	86.1^a^	91.3^a^	80.7^a^	80.7^b^	92.4	82.8^b^	84.9^a^	86.0^a^	87.9^a^	89.2	90.0^a^	83.3^ab^	75.5^ab^	82.8^a^	81.6^a^	84.7^a^	78.3	84.6^a^
	ProAct	76.8^a^	80.8	86.5^a^	91.3^a^	79.5^ab^	80.9^ab^	91.4	83.4^ab^	84.7^ab^	85.8^ab^	87.6^ab^	89.0	89.9^a^	82.5^ab^	75.0^ab^	82.4^ab^	81.5^ab^	84.7^a^	77.5	84.3^ab^
	ProAct 360	75.8^ab^	81.8	87.2^a^	91.6^a^	80.2^a^	83.1^a^	91.5	85.1^a^	86.0^a^	85.6^ab^	87.9^ab^	89.6	90.5^a^	82.4^ab^	76.8^a^	83.4^a^	82.4^a^	85.6^a^	79.0	85.2^a^
South Brazil	-	71.1^c^	79.1	82.6^b^	87.8^b^	73.7^c^	73.1^d^	90.2	76.1^d^	79.6^c^	80.9^c^	84.8^c^	86.7	86.9^b^	78.3^c^	70.2^c^	76.8^c^	76.5^c^	79.5^c^	74.7	79.7^c^
	ProAct	74.7^b^	79.8	86.0^a^	90.0^a^	76.6^b^	77.7^c^	91.7	80.1^c^	82.8^b^	84.0^b^	86.2^bc^	87.2	88.9^a^	81.4^b^	74.0^b^	80.5^b^	79.6^b^	82.5^b^	76.0	82.7^b^
	ProAct 360	76.3^ab^	81.2	86.8^a^	91.3^a^	80.5^a^	81.5^ab^	91.8	83.5^ab^	85.1^a^	86.1^ab^	88.5^a^	88.8	90.3^a^	83.8^a^	77.2^a^	83.1^a^	82.4^a^	85.4^a^	79.5	85.0^a^
Pooled-SEM		0.36	0.30	0.31	0.29	0.52	0.55	0.26	0.51	0.40	0.38	0.30	0.27	0.30	0.39	0.46	0.42	0.41	0.40	0.67	0.36
SBM source																					
North		76.0	81.5	86.6	91.4	80.1	81.6	91.8	83.7	85.2	85.8	87.8	89.2	90.1	82.7	75.8	82.8	81.9	85.0	78.3	84.7
South		74.0	80.0	85.1	89.7	76.9	77.4	91.2	79.9	82.5	83.7	86.5	87.6	88.7	81.2	73.8	80.1	79.5	82.4	76.7	82.5
	Protease																				
	-	73.2	80.4	84.3	89.6	77.2	76.9	91.3	79.4	82.2	83.4	86.4	87.9	88.4	80.8	72.8	79.8	79.1	82.1	76.5	82.1
	ProAct	75.8	80.3	86.2	90.6	78.0	79.3	91.5	81.8	83.7	84.9	86.9	88.1	89.4	82.0	74.5	81.4	80.5	83.6	76.7	83.5
	ProAct 360	76.1	81.5	87.0	91.4	80.4	82.3	91.6	84.3	85.6	85.8	88.2	89.2	90.4	83.1	77.0	83.2	82.4	85.5	79.3	85.1
Probability. *P*≤																					
SBM source		<.0001	0.018	0.002	0.001	0.0002	<.0001	0.342	<.0001	<.0001	0.0006	0.013	0.002	0.008	0.015	0.007	<.0001	0.0003	<.0001	0.262	<.0001
Protease		<.0001	0.227	<.0001	0.005	0.005	<.0001	0.857	<.0001	<.0001	0.002	0.014	0.096	0.006	0.010	<.0001	<.0001	<.0001	<.0001	0.207	<.0001
SBM source × protease		0.001	0.287	0.006	0.017	0.001	0.001	0.058	0.003	0.003	0.0004	0.010	0.348	0.039	0.0001	0.002	0.0002	0.002	0.001	0.423	0.001

Abbreviations: SBM, soybean meal.

^1^Means with different subscripts within a column differ significantly (P < 0.05).

In conclusion, the N digestibility and SIAAD of SBM in Brazil was influenced by SBM source with the South SBM sample being lower than that from the North. The second-generation protease (ProAct 360) outperformed a former developed product (ProAct) in terms of improving SIAAD. The effects were more evident in the SBM sample from the South of Brazil. It's concluded that the exogenous protease utilized in the present study can enhance the nutritional value of SBM, particularly for those with inherently low amino acid digestibility, offering economic returns for broiler producers.

## Conflict of interest statement

None.
